# An unusual case of invasive pleuritis and miliary Mycoplasma pneumonia during check‐point inhibitor therapy

**DOI:** 10.1002/rcr2.813

**Published:** 2021-07-29

**Authors:** Daniel Bird, Maninder Singh

**Affiliations:** ^1^ Respiratory and Sleep Medicine Gold Coast University Hospital Gold Coast Queensland Australia

**Keywords:** check‐point, immunotherapy, miliary, Mycoplasma, pleuritis

## Abstract

A growing body of evidence suggests that check‐point inhibitors not only increase the overall risk of infections, but, due to an altered immune response, may also result in atypical manifestations. We report a case of a 38‐year‐old man with pleuritic chest pain, dyspnoea, fevers and a dry cough receiving combination ipilimumab and nivolumab immunotherapy for metastatic melanoma. Radiological findings demonstrated a diffuse increased fluorodeoxyglucose avidity of the thoracic pleura in addition to a disseminated miliary pattern of pulmonary nodularities. A subsequent bronchoscopy was macroscopically normal with unremarkable washings. In the context of a significantly elevated Mycoplasma serology, a diagnosis of *Mycoplasma pneumoniae* pneumonia (MPP) was made. The patient was successfully treated with a course of azithromycin and amoxicillin‐clavulanic acid. We suggest an awareness of diffuse pleuritis and a disseminated miliary nodular pattern as atypical manifestations of MPP, potentially attributable to immune modulation in the context of immunotherapy.

## Graphical abstract

A novel case of disseminated miliary Mycoplasma pneumonia with invasive diffuse pleural involvement is reported. This is an atypical manifestation of a relatively common presentation likely due to altered immunity from concurrent check‐point inhibitor immunotherapy for metastatic melanoma.

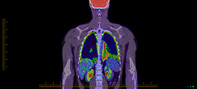

## INTRODUCTION

Whilst *Mycoplasma pneumoniae* (MP) is a common cause for respiratory infections, it is an uncommon cause of severe pneumonia in immunocompetent adults. Only 0.5%–2% of all MP pneumonia (MPP) presents with fulminant disease.^1^ Miliary pneumonia is not a known radiological manifestation of Mycoplasma infection and is often associated with more sinister causes such as tuberculosis and metastatic malignancy, which should be excluded. Extra‐pulmonary manifestations are rare and thought to be as a result of either direct invasion by the bacterium or indirectly through autoimmunity and immune complex deposition.^2^ Pleural involvement is uncommon, but when it does occur it is usually associated with parapneumonic effusions or focal pleurisy. Check‐point inhibitors are not known to increase the risk of infections beyond the general population, but may play a role in increased severity or frequency of extra‐pulmonary manifestations through immune modulation.^3^ This is a unique case of clinically significant diffuse pleuritis and disseminated miliary pneumonia due to MP infection.

## CASE REPORT

**FIGURE 1:**
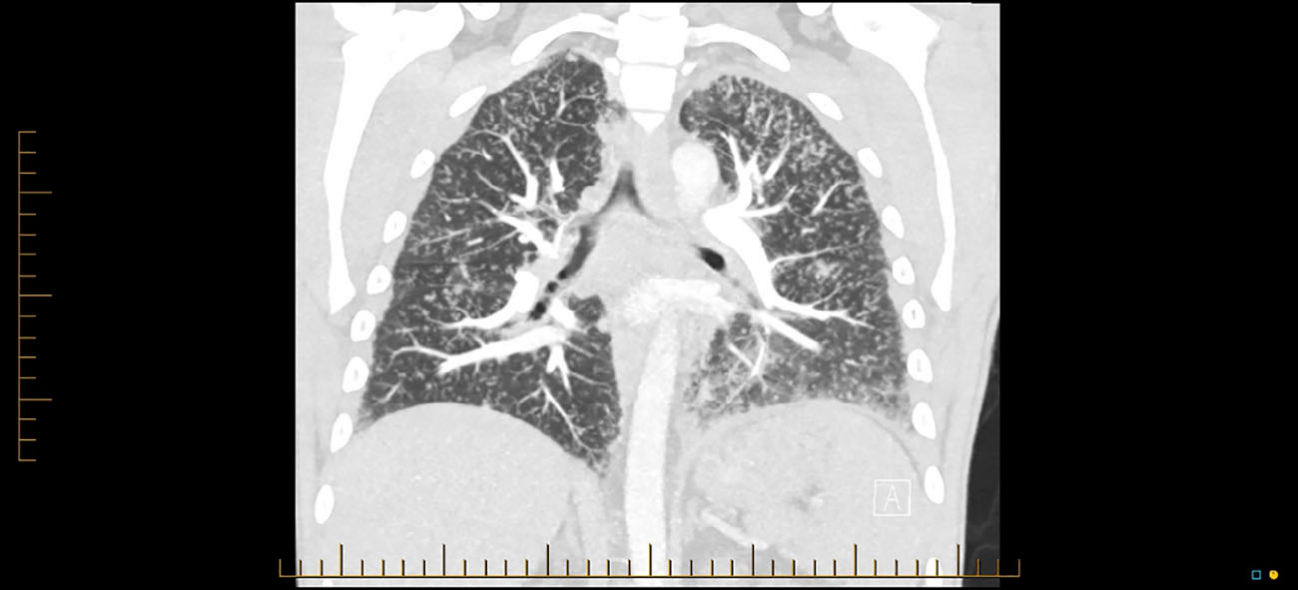
Computed tomography pulmonary angiogram with disseminated miliary micro‐nodularity

A 38‐year‐old male was admitted to hospital with a 3‐day history of fevers, sore throat, dry cough and occipital headaches. He had recently received his second cycle of ipilimumab and nivolumab for metastatic BRAF wild‐type melanoma, with brain and lung metastases. The initial differential was that of an upper respiratory tract infection but was also treated with stat oral steroids to cover for immunotherapy pneumonitis. A subsequent computed tomography (CT) brain, chest x‐ray, blood examination and nasopharyngeal respiratory polymerase chain reaction (PCR) were all unremarkable. He was treated with intravenous fluids, ceftriaxone and doxycycline. The following day he was discharged home by the medical oncology team with a diagnosis of immunotherapy‐related fevers. He represented 1 week later with new‐onset bilateral pleuritic chest pain and lethargy, as well as worsening of his original symptoms. He was now mildly hypoxic with an oxygen saturation of 92% and had fine crackles on auscultation in bilateral lower lobes. A CT pulmonary angiogram revealed extensive widespread centrilobar and perilymphatic nodularities with consolidative and ground‐glass opacities around known bilateral lower lobe metastases (Figure 1). An atypical pneumonia screen was sent off and he underwent a bronchoscopy which was macroscopically unremarkable with washings that were negative for microscopy, culture and sensitivities. His blood examination was significant for a C‐reactive protein of 51 mg/L and MP IgG/IgM antibody titre of 10,240 AU/ml. As Mycoplasma PCR testing is not routinely performed in our institution, a convalescent serological indirect particle agglutination assay was used to detect a mixture of Mycoplasma IgG and IgM. Whilst in hospital, the patient reported significant bilateral upper and lower chest wall pleurisy reaching pain scores of up to 8/10 at times.

**FIGURE 2:**
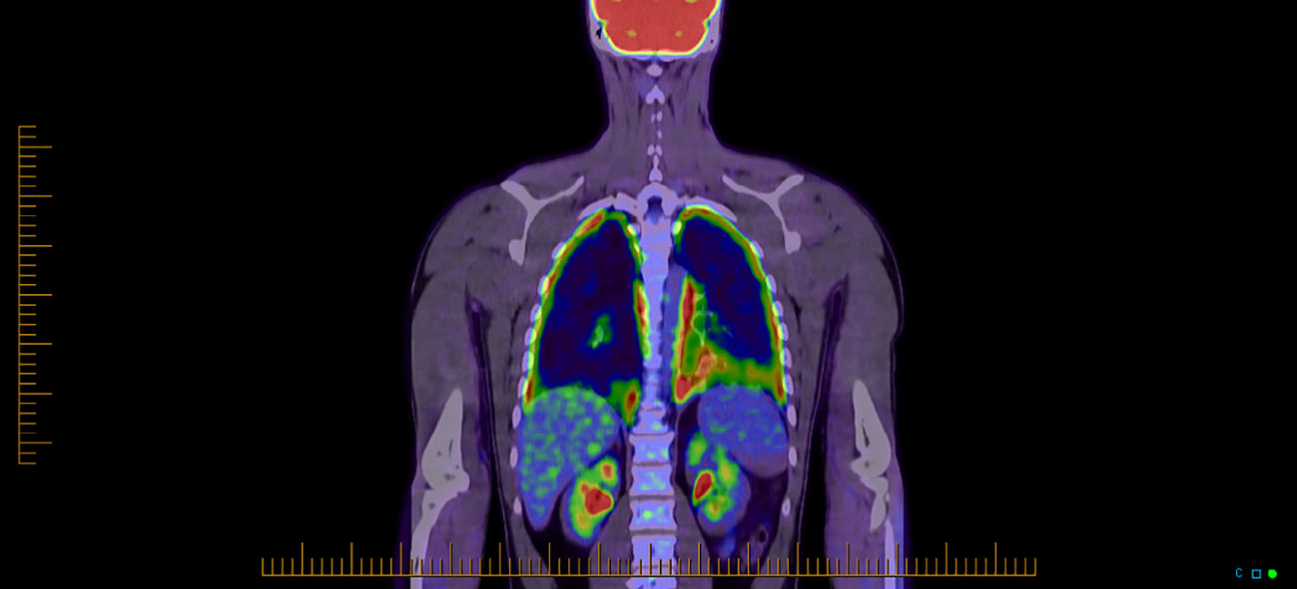
Whole‐body positron emission tomography scan with diffuse pleural fluorodeoxyglucose uptake

As a consequence of the CT findings and significantly elevated MP serology, he was diagnosed and treated for Mycoplasma pneumonia with a course of azithromycin and amoxicillin‐clavulanic acid. He achieved clinical resolution within 2 weeks, repeat convalescent Mycoplasma antibody titre was down trending to 1280 AU/ml and a CT chest at 6 weeks confirmed complete resolution of infective changes. Of interest, a surveillance whole‐body positron emission tomography scan performed the day following discharge showed diffused bilateral pleural uptake consistent with pleuritis (Figure 2). This extra‐pulmonary manifestation would explain the significant pleuritic pain he was experiencing.

## DISCUSSION

MP is a common and often commensal bacteria of the upper respiratory tract. It is a cause of both upper and lower respiratory tract infections and is a common cause of community‐acquired pneumonia. Miliary pneumonia is an uncommon radiological feature of pulmonary infections and presents radiographically as widespread micro‐nodules ranging from 1 to 3 mm in size usually in patients who are immunocompromised. Miliary micro‐nodularity should always raise a suspicion of tuberculosis or metastatic malignancy, and these should be endeavoured to be excluded. There are many non‐tuberculosis causes of diffuse micro‐nodularity of which Mycoplasma is not typically included such as Mycobacterium, hypersensitivity pneumonitis, cryptococcus, sarcoidosis, silicosis and fungal pneumonia.^4^ Classical CT findings of Mycoplasma infection include centrilobar nodularities, bronchovascular bundle thickening, ground‐glass opacites and lobar or segmental consolidation.^5^

Organizing pneumonia (OP) and immune check‐point inhibitor (ICI)‐induced pneumonia are two alternative diagnoses that have been shown to present with diffuse micro‐nodularity in the context of immunotherapy. Both these presentations differ from this reported case in that neither OP nor ICI‐induced pneumonia would have had any significant clinical response to antibiotic therapy alone, instead requiring prolonged courses of steroids to achieve clinical remission. Also, a significantly elevated initial Mycoplasma titre of >1:320 is considered diagnostic of an acute Mycoplasma infection, and the expect fall in the paired serology is also reflective of recent acute infection with an expected peak between 10 and 30 days.^6^

Extra‐pulmonary manifestations do occur but are rare and are believed to be immune mediated, rather than due to direct bacterial invasion.^2^ Parapneumonic effusions are an uncommon complication of MPP infection but are the only known significant involvement of the pleura in the literature.^2^ Significant pleural invasion or pleurisy is not known to be a feature of Mycoplasma pneumonia.^2^

A growing body of evidence suggests that check‐point inhibitors, regardless of steroid use, increases the risk of infection through its immune modulation effect and altered host's response.^3^ This immune modulation may additionally increase the risk of autoimmunity or immune complex formation resulting in an increased incidence of atypical manifestations of MPP.

In summary, we report a case of disseminated miliary pneumonia and clinically significant pleuritis with negative bronchial washings and markedly elevated Mycoplasma serology. This is in the context of concurrent check‐point inhibitor therapy for metastatic melanoma. We suggest that this is an atypical presentation of MP infection as a consequence of altered immunity.

## CONFLICT OF INTEREST

None declared.

## ETHICS STATEMENT

Appropriate written informed consent was obtained for publication of this case report and accompanying images.

## AUTHOR CONTRIBUTIONS

Both Daniel Bird and Maninder Singh had substantial contributions to the conception, design, analysis and interpretation of data for the case report. Daniel Bird was responsible for drafting the work, whilst Maninder Singh revised it critically for important intellectual content. Both gave final approval for the version to be published. Both agree to be accountable for all aspects of the work in ensuring that questions related to the accuracy or integrity of any part of the work are appropriately investigated and resolved.
